# *Stegomyia* mosquitoes in Mayotte, taxonomic study and description of *Stegomyia pia* n. sp.

**DOI:** 10.1051/parasite/2013030

**Published:** 2013-09-12

**Authors:** Gilbert Le Goff, Cécile Brengues, Vincent Robert

**Affiliations:** IRD, UMR MIVEGEC 911 Avenue Agropolis BP 64501 34394 Montpellier Cedex 5 France

**Keywords:** Culicidae, Inventory, Biodiversity, Vector, France, New species

## Abstract

Four mosquito species, including a new species of the genus *Stegomyia*, are reported from Mayotte in the western Indian Ocean. The most abundant species were *Stegomyia aegypti* and *St. albopicta*. Only one species of the *St. simpsoni* group was observed, *St. bromeliae*. The fourth species is *Stegomyia pia* Le Goff & Robert n. sp. of which the larva, pupa, male and female are here described. The larval stages of *St. pia* n. sp. are morphologically similar to *St. aegypti* but differ in the number of branches of the seta 1-X; the adult is morphologically distinct for a number of characters, for instance the scutal ornamentation. *Stegomyia pia* n. sp. is uncommon but not rare, and largely distributed across Mayotte. Its larval habitats are natural and diverse including rock pools, tree holes, and cut and severed bamboos. The biology of adults remains unknown, especially female biting behaviour. Both morphological characters and nucleotide sequences of the *ITS2* and *COI* genes indicate that this species is best placed in the genus *Stegomyia*. Dichotomous keys to the four species of Mayotte *Stegomyia* are presented for adults and fourth-instar larvae. The potential vector role of these mosquitoes is hypothesised. This paper underlines advances in knowledge of the biodiversity in the French overseas departments and territories.

## Abstract (fr)

Quatre espèces de moustiques du genre *Stegomyia* sont rapportées de Mayotte, Océan Indien Occidental, dont une nouvelle espèce pour la science. Les espèces les plus abondantes étaient *Stegomyia aegypti* et *St. albopicta*. Une seule espèce du groupe *St. simpsoni* a été observée, *St. bromeliae*. La quatrième espèce est *Stegomyia pia* Le Goff & Robert n. sp., dont la larve, la nymphe, et les adultes mâles et femelles sont ici décrits. Les stades larvaires de *St. pia* n. sp. sont morphologiquement proches de ceux de *St. aegypti* mais en diffèrent par le nombre de branche des soies 1-X; l’adulte est aisément reconnaissable grâce à de nombreux caractères, par exemple l’ornementation du scutum. *Stegomyia pia* n. sp. est une espèce peu commune sans être rare et largement distribuée sur l’ensemble de Mayotte. Ses gîtes préimaginaux sont naturels et variés, trous de rocher, trous d’arbres, bambous coupés et fendus. On ignore encore tout de la biologie des adultes, en particulier des comportements trophiques des femelles. Les caractères morphologiques ainsi que les séquences nucléotidiques des gènes *ITS2* et *COI* indiquent que cette espèce est placée dans le genre *Stegomyia*. Des clés dichotomiques de détermination des quatre espèces de *Stegomyia* de Mayotte sont proposées pour les adultes et pour les larves de stade 4. Le rôle de vecteur potentiel de ces moustiques est envisagé. Cet article contribue à la connaissance de la biodiversité de l’outre-mer français.

## Introduction

The study of the entomofauna in the Comoros archipelago commenced in earnest in 1947, under the direction of Jacques Millot working at the Muséum National d’Histoire Naturelle in Paris, who organised several scientific missions. The observations were published in a synoptic paper on the origin of the Comorian entomofauna composed of 1106 taxa grouped in 767 genera [33]. In 1973–1974, another mission of the Muséum National d’Histoire Naturelle, headed by Loïc Matile, took place in the archipelago, focusing on a small number of insect orders (Coleoptera, Lepidoptera, Homoptera, Phasmatoptera and Diptera); these collections significantly increased knowledge of the fauna [29]. During approximately the same period, Jacques Brunhes, a mosquito specialist, studied Diptera of medical and veterinary interest. Since these comprehensive studies [4–6], little work has been devoted to the morphotaxonomy of haematophagous Diptera in the Comoros archipelago. The exceptions concern Culicidae and Psychodidae Phlebotominae. They include the mention of the presence of two species belonging to the *Stegomyia simpsoni* complex [19, 20] and the description of a new species, *Zavortinkius brunhesi* Reinert, 1999, endemic in Mayotte [36]. More recently, in a synthesis of the genus *Uranotaenia* in the Malagasy biogeographic subregion, a new species was described, *Uranotaenia* (*Pseudoficalbia*) *comorensis* da Cunha Ramos & Brunhes, 2004, which is endemic in the Comoros archipelago [7]. A review of Phlebotomine sandflies was published [35], including the description of new species and subspecies.

Aedini is the largest tribe of mosquitoes in the world with 1252 species classified in 80 genera, including the genus *Aedes* Meigen, 1818. Depending on the taxonomic preferences of different authors, the taxon *Stegomyia* Theobald, 1901 [43] is considered as a subgenus of the *Aedes* genus [23] or a genus of the tribe Aedini [37]. The Mosquito Taxonomic Inventory [16] recognises 127 *Stegomyia* species and subspecies worldwide, and Huang [21] distinguishes 59 of them in the Afrotropical Region. From a medical point of view, the genus *Stegomyia* is extremely important because it groups a number of species that are vectors of human arboviruses such as dengue, chikungunya and yellow fever. Hence, different aspects of Aedini taxonomy, distribution and biology have been studied in some detail, at least with respect to most other insects.

For a complete review of the genus *Stegomyia* on Mayotte see Brunhes [4] and Bagny *et al*. [1]. Briefly, two species, *St*. *aegypti* (L., 1762) and *St*. *albopicta* (Skuse, 1895), are invasives. The third taxon, *St*. *simpsoni*, forms a complex with three Afrotropical species [19], of which two have been recorded in Mayotte (see below).

In 2011, we had the opportunity to conduct some inventories, specifically to update information on the mosquitoes of Mayotte via a project aiming to evaluate the culicid biodiversity on several islands of the western Indian Ocean [25, 39]. With these aspects in mind, the collection of immature stages was performed in natural environments, as well as in human-degraded, agricultural and urban areas, giving priority to the best preserved environments. The goal of the study was to obtain a better picture of mosquito diversity, especially those species that are potential vectors of pathogens. Here we present only the results concerning the genus *Stegomyia*. We collected material of the species *St. aegypti* (of the form *formosa*, with the exception of a single specimen of the form *aegypti*), the species *St. albopicta* and one species of the *St. simpsoni* complex, *St*. *bromeliae*. Surprisingly, we also obtained material of a *Stegomyia* species that is new for science.

## Material and methods

### Study area

Geographically, Mayotte Island, at the northern end of the Mozambique Channel, belongs to the Comoros Archipelago, which encompasses four principal islands: Grande Comore, Mohéli, Anjouan and Mayotte (in Shikomory language: Ngazidja, Mwali, Ndzouani and Maoré, respectively). These islands are all of volcanic origin. Mayotte is the oldest in the archipelago at about 8 million years. It is located 250 km W of Madagascar, 30 km SSW of Anjouan and 450 km E of the Republic of Mozambique coast. The emerged land of Mayotte reaches its maximal altitude at 660 m, including a large lagoon that covers a surface area of 1500 km^2^. The principal land features are Grande Terre, 363 km^2^ (about 39 km long and 22 km wide), Petite Terre, 11 km^2^, and about 30 islets. More than 1000 ha of natural forest remain on mostly Grande Terre, with about 15 000 ha of degraded forests in the island complex. The original forest has all but vanished and covers only 3% of the surface area. All remaining humid and semi-humid forests are located in the summital zones of the principal peaks on Grande Terre. Some reforestation projects have been undertaken, mostly with deciduous trees in the drier and southern end of Grande Terre (e.g., Choungui-Sohoa littoral forest and Dapani clump), and on some islets (e.g., Ilot M’bouzi, 84 ha). A large part of Grande Terre is now degraded in “padzas”, places scoured by erosion and exposed subsoils. Petite Terre does not have any remaining forest [11]. There is no rice cultivation, with or without irrigation. Mayotte, with its estimated 212 000 people in 2012, is densely populated (567 individuals/km^2^) and in continuous demographic increase (23 364 inhabitants in 1958).

### Inventory

Our mosquito inventory was mainly focused on collections of immature stages. Entomological prospecting was carried out by two of us (GLG & VR) with the help of “Service de la Lutte antivectorielle – Agence Régionale de Santé – Océan Indien, Mayotte”. We concentrated on natural environments, presumably with high biodiversity, but urban and agricultural areas were also prospected. The larval habitats of natural origin were mainly bamboo and tree holes, fallen coconuts and rock pools; the artificial containers were mainly abandoned wastes, piece of plastic and tyres. The first field mission took place during the end of rainy season from 22 March to 5 April 2011, and a second mission at the beginning of the next rainy season from 28 November to 2 December 2011. In total, 420 aquatic habitats with larval and/or pupal stages were inventoried. During the fieldwork, female mosquitoes were collected when biting us, although no formal human landing catch was performed.

### Systematics

Following the Aedini classification of Reinert and collaborators [37, 38] and the online catalogues of the Walter Reed Biosystematic Unit [48], the taxon *Stegomyia* is here considered at genus level [15]. Anatomical nomenclature and chaetotaxy follow Harbach and Knight [17, 18] and updated terms listed in the Anatomical Glossary of the Mosquito Taxonomic Inventory website [16]. The morphotypes of *St. aegypti* are those of McClelland [28].

### Molecular study

Molecular study targeted three genes: (i) the ribosomal internal transcribed spacer 2 (*ITS2*) gene, (ii) the mitochondrial cytochrome c oxidase subunit I (*COI*) gene and (iii) the mitochondrial NADH dehydrogenase subunit 4 (*NDH4*) gene. Molecular techniques were detailed previously for *COI* [34], and *ITS2* and *NDH4* [25]. Briefly, the following steps were successively performed: DNA extraction, gene amplification, purification and sequencing of the amplified fragment, electropherogram analysis and alignment of nucleotidic sequences. According to the objective of this study, which is not a phylogenetical analysis, a Neighbor-Joining analysis was performed for *ITS2* and *COI* using MEGA5 software [42], with the Kimura-2 parameter model and using uniform rates among sites; bootstrap values were obtained after 1000 replications. Analyses were performed on the sequences obtained in this study along with sequences from GeneBank, of which one from *Stegomyia mascarensis* was collected in 2007 at Rivière Noire, Mauritius [8] and two from the *St. simpsoni* group (i.e., *Stegomyia* subgenus *Mukwaya* [16]) collected in Uganda [32].

## Results

Of the 420 aquatic habitats with mosquitoes, *Stegomyia* were found in 159 (38%). The following four *Stegomyia* species were collected.

### Known species

#### Stegomyia aegypti

This species was the most abundant *Stegomyia* in our surveys – 70 of the 420 (17%) surveyed sites with 46 (66%) natural habitats. The most frequent habitat was cut bamboo (*n* = 13). It is a container-breeding mosquito, found in a wide range of vessel types; consequently, larval stages are very common in zones inhabited by people with a large number of anthropogenic habitats.

All 36 adults found in rural areas are referable to *St. aegypti formosa*, with only black scales on abdominal tergum I (morph F, *n* = 25) or with this tergum brindled of grey scales (morph G, *n* = 11). Contrarily, one female collected biting a man in the town of Mamoudzou (23 March 2011) had a large median patch of grey scales on the first abdominal tergum, typical of *St. aegypti aegypti* and corresponding to the morph Hap. Therefore, the two forms, *formosa* and *aegypti*, are present in Mayotte.

#### Stegomyia albopicta

This species is the second most abundant *Stegomyia* in our surveys – 57 of the 420 (14%) surveyed sites with 27 (47%) natural habitats. The most frequent habitat was cut bamboo (*n* = 9). Like *St. aegypti, St. albopicta* is a container-breeding mosquito and these two species were found in association in 24 habitats. *Stegomyia albopicta*, more so than *St. aegypti*, is able to adapt to unusual habitats such as crab holes and transient pools of water.

#### Stegomyia bromeliae

*Stegomyia bromeliae* was quite common in our surveys – 42 of the 420 (10%) surveyed sites with 38 (90%) natural habitats. All males of the *St. simpsoni* group show leg ornamentation (*n* = 21) and genitalia (*n* = 10) typical of *St. bromeliae*. All females (*n* = 19) had the ungues of the fore- and midlegs equal and toothed, a character shared by *St. bromeliae* and *St. lilii*. All of these females have leg ornamentation typical of *St. bromeliae* with one exception. This latter has fore- and midtarsomeres I and II with ornamentation typical of *St. lilii*, but the scutum and hindtarsomere V with ornamentation typical of *St. bromeliae*; and was collected as a larva in taro leaf axils with two other females of the *St. simpsoni* group typical of *St. bromeliae*, an indirect argument to consider the doubtful female as *St. bromeliae* rather than *St. lilii*.

Larval habitats were mainly leaf axils, particularly *Typhonodorum*, and less commonly banana, pineapple and taro. Other types of sites included dead leaves on the ground, bamboo pots and bamboo stumps, and tree holes in both urban and rural areas.

### *Stegomyia pia* Le Goff & Robert, n. sp.

urn:lsid:zoobank.org:act:8AA27838-E328-4E2F-BEB9-C843166F9879

Genus: *Stegomyia* Theobald, 1901

Authorship: Note that the authors of the new taxon are different from the authors of this paper; Article 50.1 and Recommendation 50A of International Code of Zoological Nomenclature [22].

Etymology: The term “pia” means “fresh” or “new” in the Shimaore language, the main language spoken in Mayotte. In addition, Pia is the first name of a stepdaughter of V. Robert.

GenBank Accession numbers for the *ITS2*, *COI* and *NDH4* sequences of two males of *Stegomyia pia* (T5 and T6: MY 648 bamboo, Convalescence, Mayotte, 2 December 2011) are included in Table 3.

This species was the least abundant *Stegomyia* in our surveys – 27 of the 420 (6%) surveyed sites.

#### Information on type series

Type locality: near the Governor House, at the locality known as “Convalescence” in the “Réserve forestière Majimbini”, located at 3.5 km west of Mamoudzou, Mayotte, France, Comoros Archipelago, northern Mozambique Channel, western Indian Ocean. Mayotte has the legal status of being a French Overseas Department and Region. Larvae and pupae were found on 12th October 2008 in a rock pool located within the forest and below a small waterfall [12°46′05.5″S; 45°11′19.1″E] at about 375 m a.s.l. (V. Robert rec.). The breeding pool was cluttered with dead leaves. Immature stages were associated with larvae of *Ur.* (*Psc*) *comorensis*. Larvae and pupae reared in the insectary gave rise to four males and one female. Visits on 26 March 2011 and 2 December 2011 did not result in the collection of any mosquito larvae in the same water pool.

Holotype: one male (1♂) ex pupa, labelled “Convalescence, 12-X-2008, Mayotte”; the male genitalia are mounted on a microscope slide in Euparal and labelled “G1”.

Allotype: one female (1♀) ex pupa, “Convalescence, 12-X-2008, Mayotte”.

Paratypes: three males (3♂♂) ex pupa, “Convalescence, 12-X-2008, Mayotte”; the genitalia of one are mounted in Euparal and labelled “G2”.

The pupal exuviae of the five adults permitted the description of the pupa.

Type series: two fourth-instar larvae, mounted on microscope slides in Euparal and labelled “trou de rocher, cascade Convalescence, 12-X-2008, Mayotte”.

Type deposition: the holotype, allotype, two paratypes, four pupal exuviae and one larva used in the descriptions of the male, female, pupa and larva are deposited in the “collection d’Arthropodes d’intérêt médical (Arim)”, Laboratory of Vector Taxonomy, IRD, 911 avenue Agropolis, Montpellier, France. One complete male paratype (1♂), one male pupal exuviae and one larva will be deposited in the Muséum National d’Histoire Naturelle (MNHN), Paris, France.

#### Other materials examined and used in the species description

- One female (1♀) ex pupa, labelled “gîte n°6, Coconi, 19 February 1956 (A. Grjebine rec.)”.
- Two fourth-instar larvae morphologically identified: one larva mounted on a microscope slide in Euparal and one larva with identification confirmed using molecular techniques; bamboo cut perpendicularly in a clump of green bamboos (labelled “MY 013”), altitude 18 m; Bouyouni (commune of Bandabroua) – 22 March 2011 (V. Robert rec.).
- One third-instar larva morphologically identified; tree hole (MY 136) at 1.8 m above the ground, in an adventitious root of a “badamier” (*Terminalia catappa* L.); [12°49′41″S–45°07′57″E], altitude 87 m; “village de gratte” (i.e., a hamlet with houses built from vegetation or sheet metal, far from urban areas, deep in the countryside, accessible via tracks, inhabited mainly during the rainy season by foreign field workers) of Bougouni (commune of Kahani) – 23 March 2011 (G. Le Goff rec.).
- One male (1♂) ex pupa and one fourth-instar larva morphologically identified; fallen coconut (MY 140); [12°49′41″S–45°07′59″E], altitude 93 m; traditional village of Bougouni (commune Kahani) – 23 March 2011 (G. Le Goff rec.).
- Four larvae (three fourth instars and one third instar) morphologically identified: two larvae mounted on microscope slides in Euparal and two larvae stage 4 with identification confirmed using molecular techniques; dry bamboo severed in longitudinal sections (MY 145) used as field fences, in a sunny place; [12°47′52″S–45°07′15″E], altitude 69 m; natural Lac Karihani (commune of Chiconi) – 23 March 2011 (G. Le Goff rec.).
- One fourth-instar larva morphologically identified; tree hole at 1.3 m high (MY 071) [12°46′22″S–45°12′19″E], altitude 212 m; Convalescence, Réserve forestière Majimbini (commune de Mamoudzou) – 26 March 2011 (G. Le Goff & V. Robert rec.).
- One third-instar larva morphologically identified; green bamboo cut at 20 cm above the ground in a bamboo clump (MY 074) [12°46′0″S–45°11′5″E], altitude 265 m; Réserve forestière Majimbini (commune of Mamoudzou) – 26 March 2011 (G. Le Goff & V. Robert rec.).
- Four larvae (two third instars and two second-third instars) morphologically identified: three larvae mounted on microscope slides in Euparal plus one larva stage 3 with identification confirmed using molecular techniques; green bamboo (MY076) cut in the same clump as MY074; Réserve forestière Majimbini (commune of Mamoudzou) – 26 March 2011 (G. Le Goff & V. Robert rec.).
- Two larvae (one fourth instar and one third instar) morphologically identified; bamboo hole (MY 078) [12°46′07″S–45°11′26″E], altitude 398 m; Convalescence, 30 m of the Governor House (commune of Mamoudzou) – 26 March 2011 (G. Le Goff & V. Robert rec.).
- Two females (2♀♀) ex pupae, morphologically identified; tree hole (MY 084) at 10 cm above the ground along the path (“sentier de Grande Randonnée”) leading to crest lines, containing about 100 mL of water heavily laden with organic materials [12°45′56″S–45°11′11″E], altitude 467 m; Réserve forestière Majimbini (commune of Mamoudzou) – 27 March 2011 (G. Le Goff & V. Robert rec.).
- One male (1♂) ex pupa; bamboo hole (MY 085), cut perpendicularly at 1 m above the ground, with clear and transparent water; [12°46′04″S–45°11′25″E], altitude 420 m; Governor House, Convalescence (commune of Mamoudzou) – 27 March 2011 (Le Goff & Robert rec.).
- Seven larvae (one fourth instar, three third instars and three second instars) morphologically identified: five larvae mounted on microscope slides in Euparal plus two third-instar larvae with identification confirmed using molecular techniques; bamboo hole (MY 086), cut perpendicularly at 1.4 m above the ground, with clear water; at 1 m of MY 084; Governor House, Convalescence (commune of Mamoudzou) – 27 March 2011 (G. Le Goff & V. Robert rec.).
- Two fourth-instar larvae morphologically identified: one larva mounted on a microscope slide in Euparal and one larva with identification confirmed using molecular techniques; bamboo hole (MY 226); [12°42′10″S–45°04′55″E], altitude 185 m; northern crest, Mont Dziani Bolé (commune of Tsamboro) – 30 March 2011 (V. Robert rec.).
- Four larvae (two fourth instars and two third instars) morphologically identified: two larvae mounted on microscope slides in Euparal and two fourth-instar larvae with identification confirmed using molecular techniques; coconut on the ground (MY 312), in a shadow place, with a very narrow opening, along a track leading to Mont Dziani Bolé [12°42′10″S–45°04′55″E], altitude 156 m; tracks towards northern crest (commune of Tsamboro) – 30 March 2011 (G. Le Goff rec.).
- One third-instar larva morphologically identified; dry bamboo (MY 328) cut at 0.5 m above the ground; [12°47′26″S–45°09′46″E], altitude 322 m; Mont Combani area (commune of Tsingoni) – 31 March 2011 (G. Le Goff rec.).
- Two larvae (one third instar and one second-third instar) morphologically identified; abandoned plastic waste hidden in dense vegetation (MY 330); with small quantity of clear rain water, near MY 328; Mont Combani area (commune of Tsingoni) – 31 March 2011 (G. Le Goff rec.).
- One fourth-instar larva morphologically identified; rock pool (MY 245), with a very narrow opening and small volume, clear rain water, in a large block of volcanic lava; in the southern part of Ilot M’bouzi, close to a sandy seashore near ocean front; Ilot M’bouzi – 01 April 2011 (G. Le Goff & V. Robert rec.).
- One fourth-instar larva morphologically identified; tree hole (MY 247), just below the cliffs leading towards the summit of Ilot M’bouzi, altitude 10 m; in tree trunk 20 cm in diameter, about 50 cm above the ground, with about 150 mL of very clear water; Ilot M’bouzi – 01 April 2011 (G. Le Goff & V. Robert rec.).
- One second-third instar larva morphologically identified; tree hole (MY 271) with very clear water; located along the footpath (“sentier de grande randonnée”) that leads to Mont Bénara; [12°52′33″S–45°09′24″E], altitude 400 m; Bénara area (commune Dembéni) – 02 April 2011 (V. Robert rec.).
- Two third-instar larvae morphologically identified; tree hole (MY 272), at 0.5 m above the ground, with rain water heavily laden with organic materials; at about 20 m of MY 271; Bénara area (commune de Dembéni) – 02 April 2011 (V. Robert rec.).
- Three fourth-instar larvae morphologically identified: two larvae mounted on microscope slides in Euparal and one larva with identification confirmed using molecular techniques; dry bamboo (MY 298) under a pile of old bamboos in the shadow of a mango tree; [12°47′45″S–45°07′16″E], altitude 48 m; natural Lac Karihani (commune of Chiconi) – 05 April 2011 (V. Robert rec.).
- Four fourth-instar larvae morphologically identified: two larvae mounted on microscope slides in Euparal and two larvae with identification confirmed using molecular techniques; rock pool (MY 623), with a lot of decomposing vegetation, in a shadow place under large trees; [12°51′30″S–45°09′19″E], altitude 150 m; track towards Mont Bénara (commune of Dembéni) – 29 November 2011 (G. Le Goff & B. Naimoudine rec.).
- One fourth-instar larva morphologically identified; decaying vegetation in a volcanic rock hole (MY 624) 3 cm in diameter with 5 or 10 mL of water; beside MY 623; track leading towards the Mont Bénara (commune of Dembéni) – 29 November 2011 (G. Le Goff & B. Naimoudine rec.).
- Four larvae (three fourth instars and one third instar) and one fourth-instar exuvia, morphologically identified: two larvae mounted on microscope slides in Euparal and two fourth-instar larvae with identification confirmed using molecular techniques; rock hole (MY 627) in the bed of a small mountain torrent (Mavingouni River); a small hole 5 cm in diameter, in the shade of large trees; [12°51′41″S–45°09′29″E], altitude 157 m; Bénara area (commune of Dembéni) – 29 November 2011 (G. Le Goff & B. Naimoudine rec.).
- Four females (4♀♀) ex pupae and two fourth-instar larvae morphologically identified: one larva mounted on a microscope slide in Euparal and one larva with identification confirmed using molecular techniques; bamboo severed lengthwise (MY 628); with about 300 mL of clear water and decomposing bamboo leaves; located along footpath leading to the Mavingouni river; [12°51′41″S–49°09′29″E], altitude 167 m; Bénara area (commune of Dembéni) – 29 November 2011 (G. Le Goff & B. Naimoudine rec.).
- Four third-instar larvae morphologically identified: two larvae mounted on microscope slides in Euparal and two larvae with identification confirmed using molecular techniques; tree hole (MY 635) at 20 cm above the ground, with clear rain water; located along the footpath (“sentier de grande randonnée”) leading to Mont Tsoungi; [12°57′32″S–45°07′48″E], altitude 307 m; Mont Tsoungi area (commune of Kani-Kéli) – 30 November 2011 (G. Le Goff & N. Pocquet rec.).
- Four fourth-instar larvae morphologically identified: two larvae mounted on microscope slides in Euparal and two larvae with identification confirmed using molecular techniques; tree hole of 10 cm diameter (MY 636), at 1.2 m above the ground, filled with about 0.5 L of rain water laden with organic materials; at 3 m of MY 635; Mont Tsoungi area (commune of Kani-Kéli) – 30 November 2011 (G. Le Goff & N. Pocquet rec.).
- Six males (6♂♂) ex pupae and four fourth-instar larvae morphologically identified; two males and two larvae were used for DNA extraction then sequencing for the *ITS2*, *COI* and *NDH4*; these two males had their genitalia mounted in Euparal with labels “648-T5” and “648-T6”; dead bamboo (MY 648), in oblique position, cut 1.3 m above the ground; [12°46′11″S–45°11′20″E], altitude 375 m; Governor House, Convalescence (commune of Mamoudzou) – 02 December 2011 (G. Le Goff & N. Pocquet rec.).

#### Adults (Figures 1 and 2)

##### Head

*Male*. Maxillary palpus composed of 5 palpomeres with white scales in basal position on palpomeres 2–5, forming complete rings on palpomeres 2 and 3 but not on palpomeres 4 and 5.

*Female*. Proboscis entirely dark, about as long as forefemur. Maxillary palpus composed of 3 or 4 palpomeres; palpomere 4 not observed; palpomere 3 with silvery scales on upper side. Clypeus and pedicel of antenna with silvery scales. A continuous line of silvery scales from interocular space to occiput through coronal suture. Ocular line with narrow silvery scales in its median part. A few black erect forked scales restricted to occiput. Lateral part of vertex with a triangular spot of decumbent silvery scales. Postgena with decumbent silvery scales.

##### Thorax

*Scutum*. Integument pale brown, mainly covered with small black scales. Scutal scales all narrow. Falciform acrostichal scales delineated by a complete median thin line that reaches prescutellar area; these scales yellowish except on anterior promontory that has silvery scales. Around median scutal fossa 2 large crescentic spots of iridescent silvery scales, with anterior side of spots slightly stretched and posterior sides widened slightly. Posterior to each large spot, a line of yellowish scales from posterior dorsocentral to lateral prescutellar areas and a small spot of yellowish supraalar scales. Prescutellar area without scales on its margin.

*Scutellum*. Large iridescent silvery scales on all three lobes, except posterior part of midlobe covered with small black scales.

*Pleura*. Integument yellowish. Several spots of silvery scales cover antepronotum, paratergite and proepisternum, form one small spot on proepimeron and subspiracular areas, and form two small spots on the meskatepisternum (one in upper and one in lower area) and the mesepimeron (one in upper and one in anterior area). No lower mesepimeral setae. No scales on postspiracular area.

*Wing*. All veins and wing fringe with black scales; female sometimes with a few silvery scales at base of costa.

*Halter*. Capitellum with light scales on lateral part and black scales on distal part.

##### Legs

*Foreleg*. Coxa with a spot of silvery scales on anterior surface. Femur mainly with black scales except apico-anterior side with light yellowish scales and knee spot of silvery scales, with protruding scales on the terminal part. Tibia entirely with black scales. Tarsomeres 1 and 2 with small basal white about 1/15 and 1/12 length of tarsomere, respectively. Tarsomeres 3–5 entirely black. Tarsomere 4 shorter than 5 in males. Foreungues equal and toothed in females, unequal and simple in male.

*Midleg*. Coxa, femur and tibia as foreleg. Tarsomeres 1 and 2 as foreleg with exception of a slightly more developed basal white spot, on basal 1/8 and 1/5, respectively. Tarsomeres 3–5 and midungues as foreleg.

*Hindleg*. Coxa as foreleg. Femur with light milky scales on proximal half and entire anterior surface; black scales, sometimes iridescent, on distal half of posterior surface; knee with a spot of silvery scales, with protruding scales on terminal part, spot slightly more developed than on fore- and midlegs. Tibia as foreleg. Tarsomeres 1–3 mainly black. Tarsomere 1 with basal ring of light yellowish scales on proximal. Tarsomeres 2–4 with basal ring of light milky scales on proximal 1/5, 1/5–1/4 and 2/3, respectively. Tarsomere 5 entirely with light milky scales. Hindungues equal and simple in female and male.

##### Abdomen

*Terga*. All terga mainly covered with black scales, with following exceptions. Basal bands of yellowish scales on terga III–VII. Large spots of silvery scales anterolaterally on terga I–VII in female and terga I–VIII in male; with triangular form on terga I–VI, with decumbent scales on basal part and protruding scales on terminal part; these spots less developed and more central on tergum VII, permitting an easier observation on dorsal face. Visible part of tergum VIII of female covered with black scales.

*Sterna*. Sterna mainly covered with light whitish scales without silver reflection. Sternum I not observed. Sterna II–IV covered with light whitish scales. Sterna V and VI with apical bands of black scales on about distal 1/4. Strenum VII mainly covered in black scales, with three round spots of light whitish scales, one basal and central and two apico-lateral. Visible part of sternum VIII in female with black scales.

##### Male genitalia (Figure 1D)

Gonocoxite wide, coniform, two times longer than wide; few thin setae on dorsomesal surface; numerous, long and strong setae and large, ridged and spatulate scales on lateral and ventral surfaces. Claspette a round tubercle, with thin setae on external part and more pronounced setae on central part, these latter structures form a right angle in their terminal half. Gonostylus short, rather thin, evenly curved; gonostylar claw with obtuse termination. Apical margin of tergum IX clearly concave in middle, with three or four thin spinulate setae on lobes.

#### Pupa

Integument light, except trumpet. Median part of terga II to V with 2–4 small round light brown patches. Setae of cephalothorax and abdomen pale, usually thin and short, except seta 1-I, 9-VII,VIII.

*Cephalothorax*. Trumpet coniform; meatus reticulate and spiculate. Seta 1-CT single; 2-CT 2-branched, thin; 3-CT single; 4,6-CT single, rigid; 5,7-CT 2-branched, thin; 8-CT 2-branched, posterior to trumpet, halfway between trumpet and mesonotum; 9-CT single; 10-CT 3-branched, short; 11-CT single, rigid, longer than 12-CT; 12-CT single.

*Abdomen*. All setae single, except following. Seta 1-I fanlike with 8–9 aciculate dendritic branches, each branch simple and subdivided; 4,5-I 2-branched, short; 6-I as long as 6-II and 7-I as long as 7-II; 1-II with 3–4 branches, rigid; 4-II with 2–3 branches, minute, supple; 1-III single, longer than other setae on segment; 2-III usually 2- (sometimes 3-) branched; 4,5-III, 3,4-IV 2-branched, short; 5-IV single, longer than 1-IV; 5-V single, longer than 1-V; 9-VII with 2–3 branches sometimes subdivided in 2, strong, straight; 9-VIII fanlike with 4–5 branches, the 2 or 3 central branches longer than external branches, strong, straight, aciculate.

*Paddle*. Inner and outer margins finely spiculate on distal half; midrib distinct whole length of paddle; seta 1-Pa single, long, brownish.

#### Larva (Figure 3 and Table 1)

##### Head

*Antenna*. Smooth, short; no longer than third length of head. Seta 1-A single, straight, inserted between 3/5 and 2/3 from base.

*Head*. As long as wide, well sclerotised; seta 4-C proximal to 6-C, divided at base usually into 4–7 thin branches; 5-C single, usually with very thin barbs on whole length or sometimes simple, 1.5 times longer than 6-C, 5-C inserted mesad of 6-C; 6-C single, smooth; 7-C single, sometimes aciculate on basal third; 8,9,10-C single; 11-C 3-branched from basis; 12-C 2-branched; 13-C single; 14-C with 3–4 branches; 15-C with 2–3 branches; mentum with 11–12 teeth in each side of median tooth, 7 mesal teeth closeset, 4 lateral teeth slightly separated.

##### Thorax

Seta 1-P with 3–4 branches, long; 2-P single; 3-P 2-branched; 4-P 3-branched; 5,7-P 2-branched, 6-P single, all three long and aciculate; 5-P slightly longer than 6-P; 8-P with 4–5 branches, short; 9-P single or 2-branched; 10-P single, long, barbed; 11-P with 2–3 branches, short, supple; 12-P single, long, barbed.

Seta 0-M a small tuff, about 5-branched; 1-M with 3–5 branches; 2,3,4-M single; 5-M single, strong, long, aciculate; 6-M 2-branched, strong, aciculate; 7-M single, aciculate; 8-M with 3–4 branches; 9-M 2-branched, long; 10,12-M single, long, aciculate; 14-M usually with 4–6 branches. Basal tooth of meso- and metapleural setal groups strong, prominent, hooked, strongly sclerotised.

Seta 1-T a small tuff, about 4-branched; 2-T single; 3,4-T 2-branched; 5-T 3-branched; 6-T single; 7-T 4- (sometimes 3-) branched, strong, long, barbed; 9-T 2-branched, long, barbed; 10-T single, long, barbed; 11-T single, thin; 12-T reduced; 13-T with 4–5 branches, rigid.

##### Abdomen

Seta 6-I usually 3-branched; 7-I single. Seta 6-II to 6-V usually 2- (rarely 3-) branched. Seta 6-VI with 1–2 branches.

*Segment VIII*. Seta 1-VIII with 3–5 branches; 2-VIII single, 0.90 length of 1-VIII; 3-VIII with 4–7 branches, aciculate; 4-VIII single; 5-VIII usually with 3–5 branches, barbed; comb usually of 8–10 scales (mode 8, range 7–11, *n* = 50 observations), scales sclerotised, toothlike, in single slightly curved line; each scale with ovoid corpus, edged laterally with 4–6 spicules progressively more strongly developed distally, with three large median apical spicules giving a trident appearance to each comb scale.

*Segment X*. Saddle incomplete ventrally, sclerotised, with rows of thin spicules, especially on dorsocaudal margin; seta 1-X 1.5 times as long as saddle, with usually 2–4 barbed branches (mode 3, range 1–7, *n* = 62 observations, among which one unique observation of a single seta on left side and 2-branched on right side of one specimen); 2-X 2-branched, simple; 3-X single, simple; ventral brush (4-X) with five pairs of setae, each seta 2-branched, inserted on grid.

*Siphon*. Sclerotised, mean siphonal index 2.5 (standard deviation 0.4, range 2.0–3.1, *n* = 19 observations). Pecten with usually 12 spines (mode 12, range 8–23, *n* = 54 observations), each spine with a main denticle and three secondary denticles on basal and proximal side; sometimes last spine with a 4th denticle on distal side; in 15% of observations, last pecten spine (rarely last two pecten spines) separated from other spines. Seta 1-S with 2–3 branches, smooth, inserted near distal pecten spine.

#### Diagnosis with regard to larval stage in other *Stegomyia* present in Mayotte

A quick examination of larval specimens allows separation of *Stegomyia* in two groups, the first group with a large, prominent and strongly sclerotised tooth on the plate that supports the meso- and metathoracic pleural setal groups (*St. pia* and *St. aegypti*), and the second group with these teeth poorly or normally developed (*St. albopicta* and *St. bromeliae*). The distinction between *St. pia* versus *St. albopicta* and *St. bromeliae* at larval stage, in addition to the aspect mentioned above, is simple using the comb scales and the distribution of the denticles on the pecten spines. On the other hand, the distinction between *St. pia* versus *St. aegypti* is much more difficult. Three morphological characters are relevant, none which are diagnostic. The general aspect of the larval setae is a means to separate these species, with setae largely developed for *St. pia* and notably smaller and less dense in *St. aegypti*. A character easy to observe, although not diagnostic, is the number of branches of the saddle seta 1-X: usually more than two-branched (range 1–7) in *St. pia* and usually two-branched (range 1–3) in *St. aegypti* (Figure 4). Another character is the number of branches of seta 4-P (usually with 2–3 branches [range 1–3] in *St. pia* and usually single [range 1–3] in *St. aegypti*). Using these three characters, the identification of *St. pia* larvae was possible, and indeed, confirmed by molecular techniques for 86% of specimens (Table 2).

**Table 1.** Number of branches for setae of fourth-instar larvae of *Stegomyia pia* n. sp. Mode (range) obtained from counts made on at least 10 larvae.

	Head C	Thorax			Abdominal segments		
Seta No.		P	M	T	I	II	III
0	–	–	5 (3–12)	–	–	1	1
1	1	3 (2–6)	4 (3–5)	4 (3–8)	5 (4–9)	5 (4–8)	4 (3–7)
2	–	1 (1–2)	1 (1–2)	1 (1–2)	4 (3–8)	3 (2–10)	3 (3–8)
3	1	2 (1–3)	1	2 (2–4)	1 (1–2)	1	1
4	5 (4–10)	3 (1–3)	1	2 (1–4)	3 (2–4)	3 (2–6)	1 (1–3)
5	1	2 (2–3)	1	3 (2–7)	5 (4–8)	5 (3–8)	4 (3–8)
6	1	1	2 (2–3)	1	3 (2–3)	2 (2–3)	2
7	1	2 (1–3)	1	4 (3–8)	1	3 (2–6)	3 (3–6)
8	1	5 (3–6)	3 (3–4)	4 (3–8)	–	2	2 (1–3)
9	1 (1–2)	1 (1–2)	2	2 (1–2)	2 (1–3)	2 (1–3)	3 (1–3)
10	1	1	1	1	1	1	1
11	3 (2–4)	2 (2–4)	1	1 (1–2)	5 (4–10)	1	1
12	2 (2–5)	1	1	1	–	1	1 (1–2)
13	1 (1–2)	–	3 (3–7)	5 (3–9)	5 (4–12)	3 (3–11)	3 (3–10)
14	4 (2–6)	2 (2–3)	4 (4–1 3)	–	–	1	1
15	2, 3 (2–4)	–	–	–	–	–	–
	Abdominal segments						
Seta No.	IV	V	VI	VII	VIII	X	S
0	1	1	1	1	1 (1–2)	–	–
1	4 (3–5)	4 (3–4)	3 (3–7)	3 (3–4)	3 (3–5)	3 (2–7)	3 (2–3)
2	3 (2–7)	3 (2–6)	3 (2–5)	3 (2–3)	1	2	1
3	1	1	1	1 (1–2)	4 (4–7)	1 (1–2)	–
4	1 (1–3)	1 (1–3)	1 (1–2)	1	1		–
5	4 (3–8)	4 (3–6)	4 (3–4)	3 (3–4)	3 (3–6)	–	–
6	2	2	1 (1–2)	2 (2–4)	–	–	1
7	3 (2–5)	3 (2–4)	3 (2–4)	1 (1–2)	–	–	–
8	1 (1–2)	1 (1–2)	2 (1–3)	4 (2–6)	–	–	1
9	3 (1–4)	2 (1–4)	2 (1–3)	2 (2–5)	–	–	1
10	1	1	1	1 (1–2)	–	–	–
11	1	1 (1–2)	1	1	–	4a	2
12	1 (1–2)	1	1	1	–	4b	2
13	3 (3–4)	3 (3–4)	4 (3–4)	3 (2–5)	–	4c	2
14	1	1	1	–	1 (1–2)	4d	2
15	–	–	–	–	–	4e	2 (1–2)

**Table 2.** Identification of larvae of *Stegomyia pia* n. sp. and *St*. *aegypti* using morphological characters versus *ITS2* and *NDH4* sequences. The percentage of concordance for the two data sets is provided between parentheses. All fourth-instar larvae were collected on Mayotte during 2008–2011, mainly in natural habitats.

		Morphological observations			
		*St. pia* n. sp.	Doubtful	*St. aegypti*	Total
Molecular sequences	*St. pia*	18 (86%)	0	3	21
	*St. aegypti*	7	1	14 (64%)	22

**Table 3.** Specimens used for DNA sequencing, and GenBank accession numbers.

			GenBank accession number		
Species of *Stegomyia*	Origin	Date of collection*	*ITS2*	*COI*	*NDH4*
*St. aegypti*	Mayotte 26	01/04/2011	KF135505	KF135492	KF135500
	Mayotte 27	22/03/2011	KF135506	KF135493	KF135501
*St. albopicta*	Mayotte 76	22/03/2011	KF135507	KF135494	
	Mayotte 78	22/03/2011	KF135508	KF135495	
	Greece		AY741376	AY748238	
	China		AF305554	JQ728300	
*St. bromeliae*	Mayotte 71	22/03/2011	KF135509	KF135496	
	Mayotte 72	23/03/2011	KF135510	KF135497	
*St. mascarensis*	Mauritius	2007	KF135504	HQ623047	
*St. pia* n. sp.	Mayotte 21	02/12/2011	KF135511	KF135498	KF135502
	Mayotte 22	02/12/2011	KF135512	KF135499	KF135503
*St. simpsoni* gr.	Uganda A		AF158232		
	Uganda NA		AF158230		

* Day/month/year (only for mosquitoes sequenced in the present study).

Neighbor-Joining trees were drawn using *ITS2* and *COI* sequences of five species of *Stegomyia* distributed in the western Indian Ocean (Figure 5). The specific status of these species was strongly supported.

#### Justification for placing *St. pia* within the genus *Stegomyia*

Following Huang [21], *Stegomyia* is characterised by the following combination of characters: adults (both sexes). Vertex with all broad, flat decumbent scales, erect forked scales not numerous, restricted to occiput; maxillary palpus of male not particularly short, more than 0.5 length of proboscis, five-segmented, palpomeres 4 and 5 subequal, slender and with only a few short setae, total length of apical two palpomeres not very short, at least 0.4 length of the remaining palpomeres; in females about 0.14–0.32 length of proboscis, with three or sometimes four palpomeres, palpomere 4 minute when present; maxillary palpus with white scales; acrostichal setae absent; prespiracular setae absent; postspiracurar setae present; postprocoxal membrane without scales; scutum with all, or mainly with narrows scales; scutellum with broad scales on all lobes; mesopostnotum bare; wing with narrow plume scales; hindtarsus with basal white band on at least one tarsomere. Male genitalia. Aedeagus strongly toothed; claspette well developed, with numerous setae; gonostylar claw present. Female genitalia. Insula longer than broad, with minute setae and 2–10 larger setae on apical 0.25–0.50; cerci short and broad; three spermathecal capsules, one larger than the other two. LARVAE. Seta 4-C well developed, branched, closer to 6-C than 5-C, inserted cephalad and mesad of 6-C; 4-C and 6-C inserted cephalad of antennal base; 6-C inserted cephalad of 5-C and 7-C; seta 12-I not developed; seta 2-VIII distant from 1-VIII; comb scales in a single row; ventral brush (seta 4-X) with four to five pairs of setae on grid; without precratal setae. In addition of this combination of characters, all the supplementary characters listed in the Mosquito Taxonomic Inventory website [16] that distinguish the genus *Stegomyia* from other genera of subfamily Culicinae were verified (except those related to female genitalia, not observed in the present study). Therefore, we confirm the placement of *St. pia* within the genus *Stegomyia*.

#### Justification for placing *St. pia* in subgenus *Stegomyia* (= Aegypti Group of Huang [21])

Following Huang [21], the diagnosis for the Aegypti Group is based on the following combination of characters: Maxillary palpus with white scales; scutum with dorsocentral setae; scutum with a distinct patch of broader crescent-shaped white scales on the fossa; subspiracular area with broad white scales; postspiracular area without scales; paratergite with broad white scales; scutellum with broad white scales on all lobes; white knee-spot present on all femora (except in *mascarensis*); all tibiae anteriorly dark, without any white band; hindtarsus with a basal white band on tarsomeres 1–4; and hindtarsomere 5 all white. In addition of this combination of characters, all the supplementary characters listed in the Mosquito Taxonomic Inventory website [16] that distinguish the subgenus *Stegomyia* from other subgenera of genus *Stegomyia* were verified, except two characters. Indeed, *St. pia* exhibits larger foreungues of males without tooth and larval seta 1-VIII slightly longer than 2-VIII. However these characters are considered of minor importance. Therefore, we confirm the placement of *St. pia* within the subgenus *Stegomyia*.

#### Distribution, bionomics and potential vector role

On the basis of current information, *St. pia* is considered endemic to Mayotte. It is an uncommon species although not rare.

It was collected across the elevational range from 20 to 450 m (Figure 6). It is distributed in the northeast (Bouyouni) and west (Lac Karihani) coastal plains, as well as in forested areas along the paths leading to upper elevational zones (Monts M’tsapéré, Combani, Bénara). Our records indicate that it is broadly distributed across Grande Terre, from the foothills of Mont M’tsamboro in the northwest to the surroundings of Mont Choungui in the south. It was also collected on a small-uninhabited islet (Ilot M’bouzi) where introduced lemurs and rats are numerous. We do not have any records of it on Petite Terre, but its presence there is possible.

The distribution of *St. pia* encompasses the remaining forests on Grande Terre in reserve zones, which are the last remaining natural habitats, slightly or non-degraded by human activities. Although not strictly dependant on natural forested environments, this species needs woody zones. In other words, its geographical range excludes urban areas, villages and cultivated zones. However, its verified presence in habitats close to inhabited buildings is confirmed, as observed in March 2011 in Bouyouni within 30 m of houses and in the “centre” of Bougouni, a traditional village in the commune of Kahani.

The larval habitats are of natural origin: bamboo and tree holes, fallen coconuts and rock pools (Table 4). More specifically, the vast majority of these sites are associated with vegetation, particularly bamboos and trees, which constituted about 75% of the documented locations. In the case of bamboos, the sites varied from being green and standing or dry and on the ground. In most cases, cut bamboos occurred in the peripheral area of the clump and in the shade; on a few occasions, *St. pia* was also collected in bamboos severed in longitudinal sections, used as field fences and exposed to the sun.

**Table 4.** Distribution of the immature stages of *Stegomyia pia* n. sp. based on season and the type of habitats collected during the 2011 field surveys.

Season	Rock pools	Cut bamboos	Tree holes	Fallen coconuts	Abandoned wastes	Total
Beginning of rainy season (October–November)	3	2	2	0	0	7
End of rainy season (March)	1	10	6	2	1	20
Total	4	12	8	2	1	27

Tree holes were mainly of small diameter, about 10 cm, at various heights, ranging from the lower portion of the tree (10–20 cm high) up to at least 2 m on the trunk. Tree species were not identified, except in one case for a hole at 1.8 m high, in an adventitious root of a “badamier” (*Terminalia catappa* L.). These places ordinarily contain 20–100 mL of water (max 500 mL), heavily laden with organic materials, and often a dark coffee colour.

Rock pools are important habitats, during the dry season, when ovipositioning in different types of vegetation disappears because of the lack of water. At such sites, there is little water, and the openings to the sources are notably small, 2 or 3 cm in diameter, which presumably reduces evaporation. These types of small cavities are common in the volcanic rock, particularly in the bed of small mountain torrents and downstream from waterfalls where there is shade and a constant source of rain and river water. The presence of plant matter is common. The only exception to known sites for this species in natural situations is one found in abandoned plastic waste hidden in dense vegetation on the track leading to Mont Combani. This habitat can be considered as “natural” with regard to the availability of rain water, and the co-occurrence at the same site of the larvae of *Ur.* (*Psc*) *comorensis*, a culicid species typical of forest.

Larval development is slow, at least with regard to other *Stegomyia* encountered. Adults reared from fourth-instar larvae and pupae were obtained after 3–4 days in the insectary in only five sites among 19 (26%). With *St. aegypti*, for instance, this proportion was much higher (39/70 = 56%).

The bionomics of adults are unknown, including the trophic and resting preference of females and their longevity.

#### Associated culicid fauna

In 67% of cases (18/27), *St. pia* shared its larval habitats with other mosquito species that ordinarily occupy habitats of small size (stenotrophe mosquitoes) in woody zones on Mayotte. In decreasing order, the following have been found in association with *St. pia* (number of occasions in parentheses): *St*. *aegypti* (7), *Eretmapodites subsimplicipes* Edwards, 1914 (6), species of the *Zavortinkius monetus* group (Edwards, 1935) (5), *Ur. comorensis* (4), *Orthopodomyia* spp. (4), *Culex horridus* Edwards, 1922 (3), *Cx*. *carleti* Brunhes & Ravaonjanahary, 1971 (2), *Cx*. *nebulosus* Theobald, 1901 (2) and *St*. *albopicta* (1). When examining the types of places *St. pia* inhabits, there is no obvious preferential association, although it is more often collected with *Er. subsimplicipes* and *Cx. carleti* in cut bamboos and with species of the *Za. monetus* group and *Or*. *comorensis* in tree holes. Interestingly, larvae of *St. pia* have been observed as companion larvae with the two most frequent *Stegomyia* in Mayotte, *St. aegypti* (seven occasions, two of which were confirmed by molecular techniques for both species, at Lac de Karihani and Bouyouni) and *St. albopicta* (one occasion at Bouyouni).

### Key to the adults of the *Stegomyia* of Mayotte (Figure 7)

1. Scutum with a median-longitudinal stripe of white or yellow scales .......... 2
  - Scutum with a pair of submedian-longitudinal white stripes, but without median-longitudinal white stripe .......... 3
2. Scutum with a median-longitudinal white stripe; clypeus without white scales patches .......... *albopicta*
  - Scutum with a narrow median-longitudinal yellow stripe; clypeus with white scales patches .......... ***pia* n. sp.**
3. Scutal fossa white patch narrow at base; white bands in moon crescent (or in lyre) of which the posterior part goes on in two straight white stripes .......... *aegypti*
  - Scutal fossa white patch broad at base along scutal margin; scutum with two large white and triangular spots in anterolateral position; posterior of scutum with four light stripes (usually white, sometimes yellowish) .......... subgenus *Mukwaya*

### Key to the fourth-instar larvae of the *Stegomyia* of Mayotte

1. Spines of the meso- and metathoracic pleural setal groups large, prominent, brown, strongly sclerotised and sometimes hooked .......... 2
  - Spines of the meso- and metathoracic pleural setal groups poorly or normally developed, of pale colour .......... 3
2. Thoracic setae well developed, giving a strongly hairy appearance; seta1-X usually with three or more branches .......... ***pia* n. sp.**
  - Thoracic setae poorly or normally developed; seta 1-X typically single or two-branched .......... *aegypti*
3. Pecten spines with spicules on one side, at the base of ventral side; ventral brush composed of single setae .......... *albopicta*
  - Pecten spines with spicules on the both sides; ventral brush composed of two-branched setae .......... subgenus *Mukwaya*

## Discussion

*Stegomyia aegypti* is a polymorphic species. Mattingly [30] described two subspecies based on overall body colour and the extent of white scaling on the first abdominal tergum. According to Mattingly, (i) the darker and presumably ancestral *St. aegypti formosa* is confined to Africa, where it prefers natural, as opposed to artificial, larval habitats and is predominantly zoophilic in its biting behaviour, and (ii) the lighter *St. aegypti aegypti* is an invasive taxon distributed throughout the tropics outside Africa, where it is strongly anthropophilic and endophilic. The *queenslandensis* form is the palest form of the subspecies *aegypti* (see also [28]). More recently these two subspecies were observed nearly in sympatry in some places along the coast of East Africa, including some localities of Kenya ([41] and other references herein), where *aegypti* inhabits in villages and *formosa* in surrounding forests, with little gene flow between the two subspecies [3]. The Neighbor-Joining trees (Figure 5) indicate the close proximity of *St. aegypti* and *St. mascarensis*, in agreement with their placement in the subgenus *Stegomyia* (the Aegypti Group of Huang [21]).

The first record of *St. aegypti* in Mayotte was in 1942 (as *Aedes fasciatus*) [24] and regularly observed thereafter [1, 2, 4, 13, 27]. Brunhes [4] reported the presence of several forms from *formosa* to *queenslandensis* in the Comoros Archipelago and, indeed, in the Collection Arim (Arthropodes d’intérêt medical at IRD, Montpellier) all the *St. aegypti* females collected during the period 1955–1971 were *formosa* (*n* = 18: 15F + 2G + 1H-) on Mayotte, *formosa* (*n* = 7: 3F + 2G + 1H-) and *aegypti* (*n* = 1: 1H) on Mohéli, and *formosa* (*n* = 1: 1F), *aegypti* (*n* = 8: H and Hap) and *queenlandensis* (*n* = 3: J1, K2 and N2) on Anjouan. Bagny *et al.* [1] reported the presence of *formosa* on Mayotte only in 2007. Our survey confirms that *St. aegypti formosa* is the only subspecies of this taxon largely distributed across the island. Further studies are needed to evaluate the importance of the single female *St. aegypti aegypti* (of Hap type) collected biting a man on 23 March 2011 in the town of Mamoudzou, which is the most urbanised area on Mayotte.

This species is suspected to have been the major vector of a supposed Dengue epidemic that raged across the island, especially in Dzaoudzi, in 1943 [27].

*Stegomyia albopicta*, the “Asian tiger mosquito”, is native to Southeast Asia. Initially described from the Indian Peninsula since the early 20th century this species has been observed in Madagascar [10], the Seychelles [31, 47], Mauritius (de Grandpré et de Charmoy in [14]) and La Réunion [10]. For the last 30 years, this species has progressively adapted to human environments and has spread from its native area. Like *St. aegypti*, it is now distributed on every continent, Antarctica excluded. The first record of *St. albopicta* in Mayotte was in November 2001 for immature stages found in old water-filled tyres at Mamoudzou, the biggest city, prefecture and harbour [12]. That this species was “recently introduced” in Mayotte is interestingly confirmed by its close proximity in the Neighbor-Joining trees (Figure 5) with specimens from Greece, where the species was also recently introduced [40], and the distance to specimens from China which lies within the native area of the species. In 2005–2006, a Chikungunya epidemic hit Mayotte, affecting 37% of the human population [26]. The presence of *St. albopicta* on the island probably played an important role in the emergence of this infectious disease. The vector implicated in the transmission of the disease has not been precisely identified, although it was most probably *St. aegypti* and *St. albopicta* [1]. An outbreak of Dengue 3 occurred on Mayotte in March 2010 [26], and, once again *St. aegypti* and *St. albopicta* were the suspected vectors.

Based on our findings in urban areas *St. aegypti* populations are being replaced by *St. albopicta*, which confirms the observations of other researchers [1, 2, 8]. The increasing urbanisation of the island seems to considerably favour the presence of the invasive *St. albopicta*.

The subgenus *Mukwaya* (previously the Simpsoni Group [16]) includes 10 species in the Afrotropical region [21], with three of these forming the *St. simpsoni* complex: *St. simpsoni* (Theobald, 1905) [44], *St. lilii* (Theobald, 1910) [45] and *St. bromeliae* (Theobald, 1911) [46]. These three species were formerly placed in synonymy [9], but all have been restored to specific status [19, 21]. According to Huang [19, 20], *St. simpsoni* is only distributed in the Republic of South Africa and Swaziland, *St. lilii* in West Africa, Uganda, Sudan and Ethiopia, and *St. bromeliae* with a larger distribution forming a triangle between Guinea, Ethiopia and South Africa. *Stegomyia simpsoni* has not been documented in the Comoros Archipelago [20]. *Stegomyia lilii* was noted in the “Comores Is.” (Table 2 in [19]) and on Mayotte [1]. *Stegomyia bromeliae* was noted in the “Comores Is.” (Table 2 in [19]) and on Grande Comore (Figure 1 in [20]). Females of *St. simpsoni* are easily distinguished by having the fore- and midlegs with simple ungues, whereas *St. bromeliae* and *St. lilii* have the fore- and midlegs with toothed ungues and different leg ornamentation [19, 20]. Males of these three species differ by genitalia characters [21]. The first record of the subgenus *Mukwaya* on Mayotte was in 1955 by Brygoo and Escolivet at Coconi village [13], with regular records thereafter [4]. Today, individuals of this subgenus are common on the island. In line with published literature [19, 20], our study confirms that *St. simpsoni* is not present on Mayotte. Conversely, our data suggests that *St. bromeliae* is the only species of the *St. simpsoni* complex present on Mayotte; this view is today shared by Dr. Yiau-Min Huang (pers. com.). The considerable morphological similarity between *St. bomeliae* and *St. lilii* may explain possible confusion between these two species [32]. Interestingly, the nucleotide sequences of *St. bromeliae* in Mayotte are closer to the anthropophilic population of the subgenus *Mukwaya* in Uganda than to the non-anthropophilic ones (see [32]), suggesting that the anthropophilic mosquitoes in Uganda belong to *St. bromeliae*. Curiously, species of the subgenus *Mukwaya*, specifically *St. bromeliae*, are not known on Madagascar, some 250 km from Mayotte.

**Figure 1.**
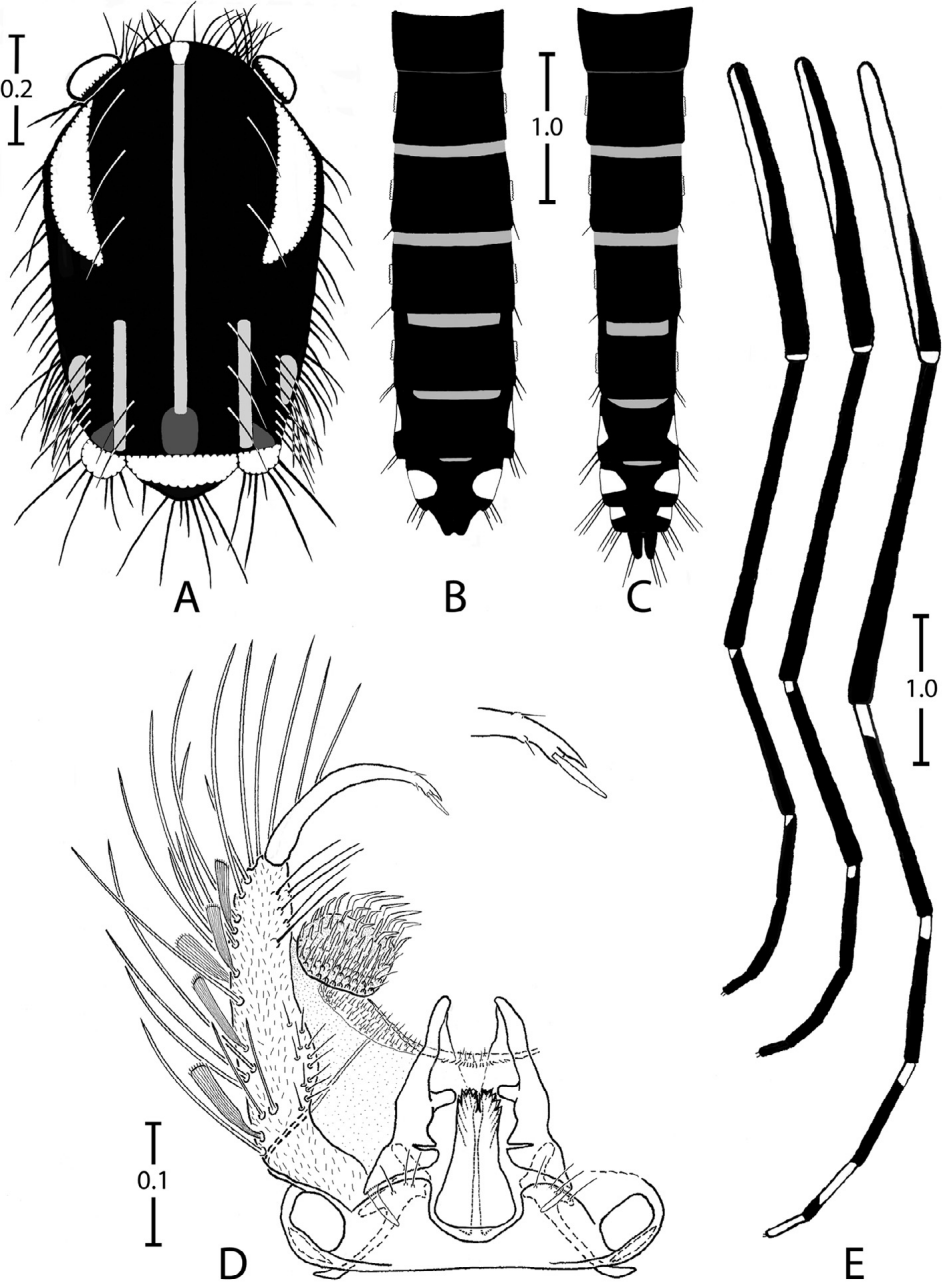
*Stegomyia pia* n. sp., adults. A, thorax of a female (dorsal view); B, abdomen of a female (dorsal view); C, abdomen of a male (dorsal view); D, male genitalia; E, fore-, mid- and hindlegs of a female (anterior view). Scales in mm.

**Figure 2.**
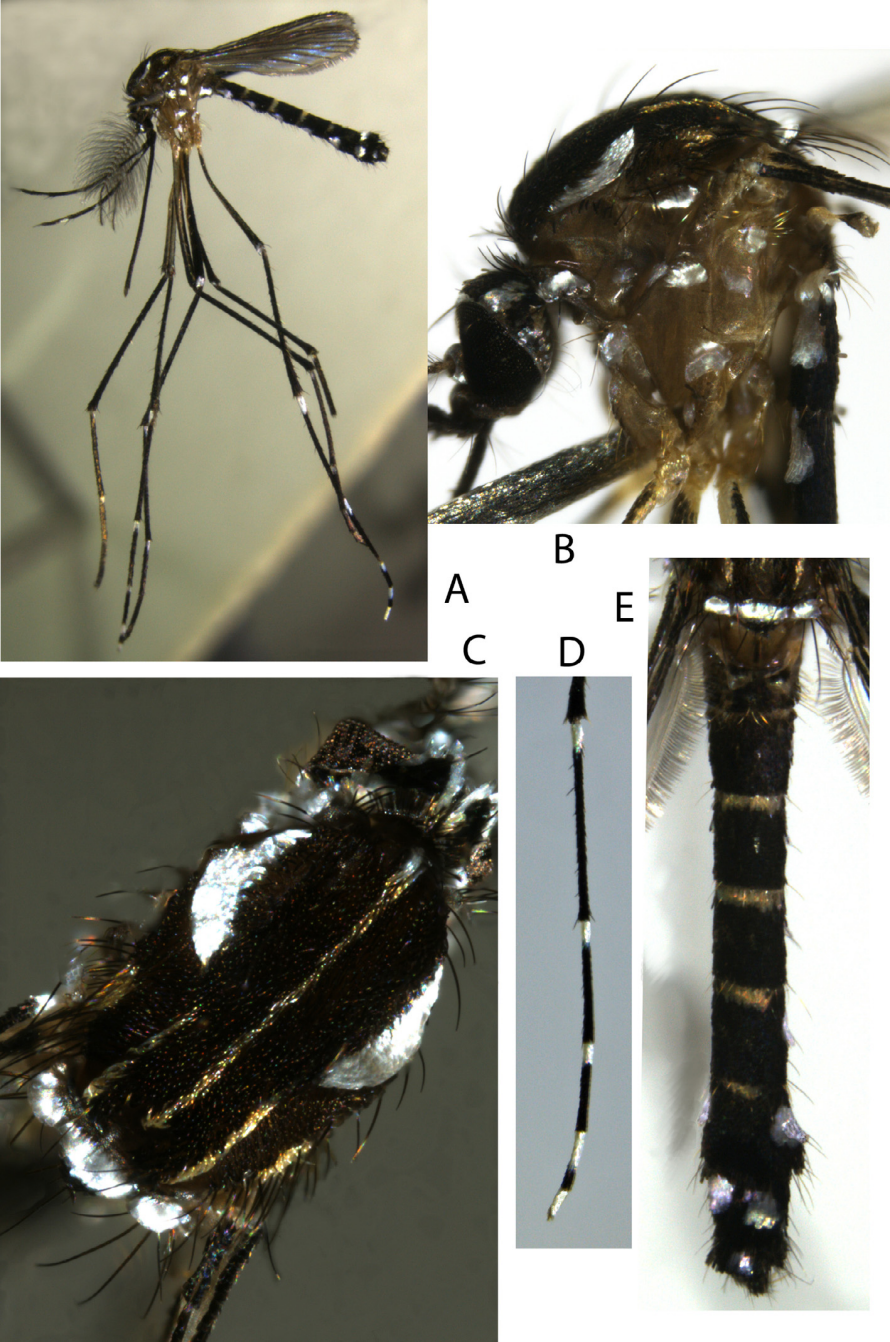
*Stegomyia pia* n. sp., adults. A, a male (lateral view); B, thorax of a female (lateral view); C, scutum of a female (dorsal view); D, hindtarsus of a female (lateral view); E, abdomen of a male (dorsal view). (Male specimen MY 085 and female specimen MY 628.)

**Figure 3.**
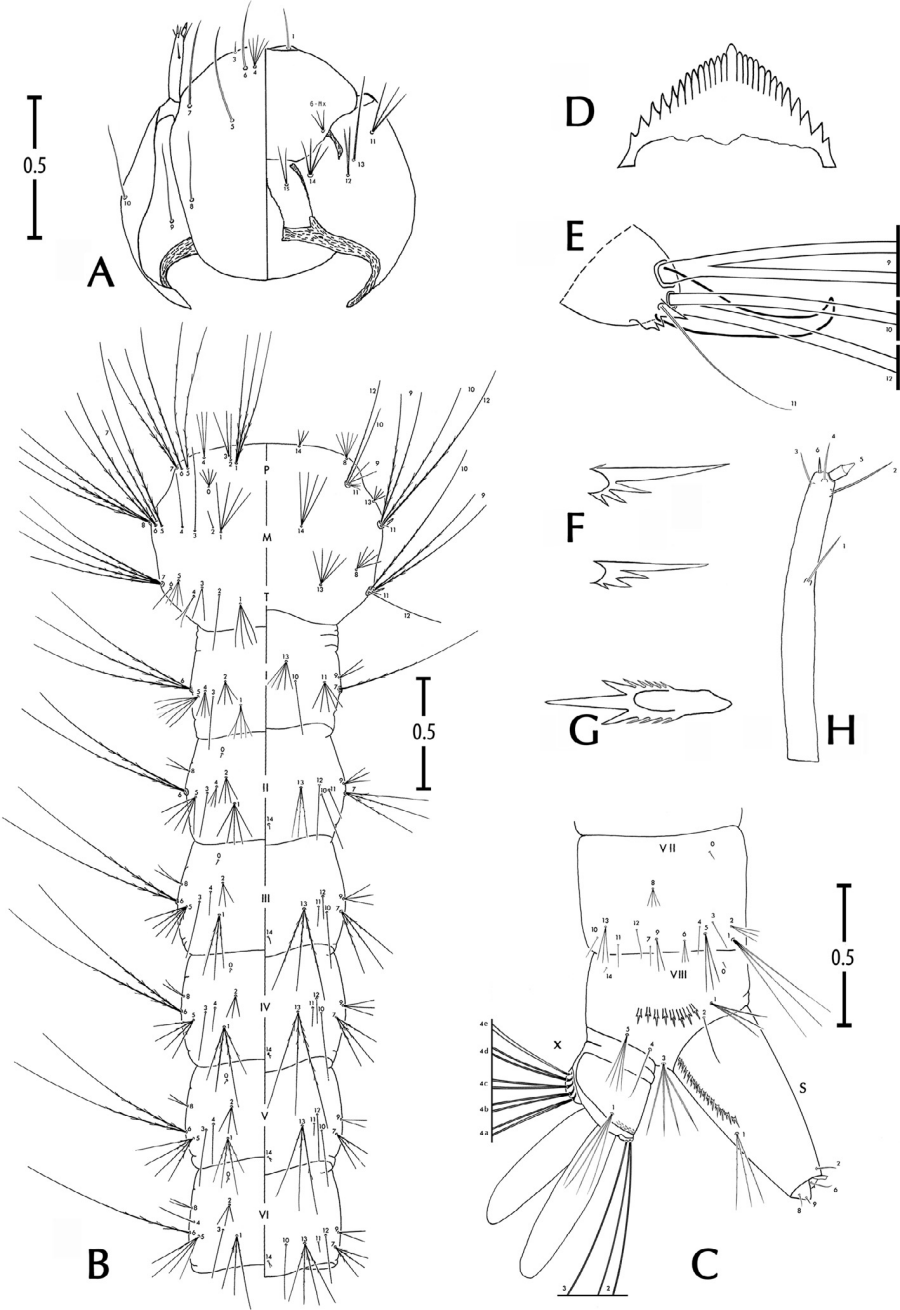
*Stegomyia pia* n. sp., fourth-instar larva. A, head (left, dorsal view; right, ventral view); B, thorax and abdomen, segment I–VI (left, dorsal view; right, ventral view); C, terminal part of the abdomen, segments VII–X (lateral view); D, dorsomentum; E, mesothorax, detail of the tooth; F, pecten, detail of subbasal and subterminal spines; G, comb, detail of a median scale; H, antenna. Scales (for A, B and C only) in mm.

**Figure 4.**
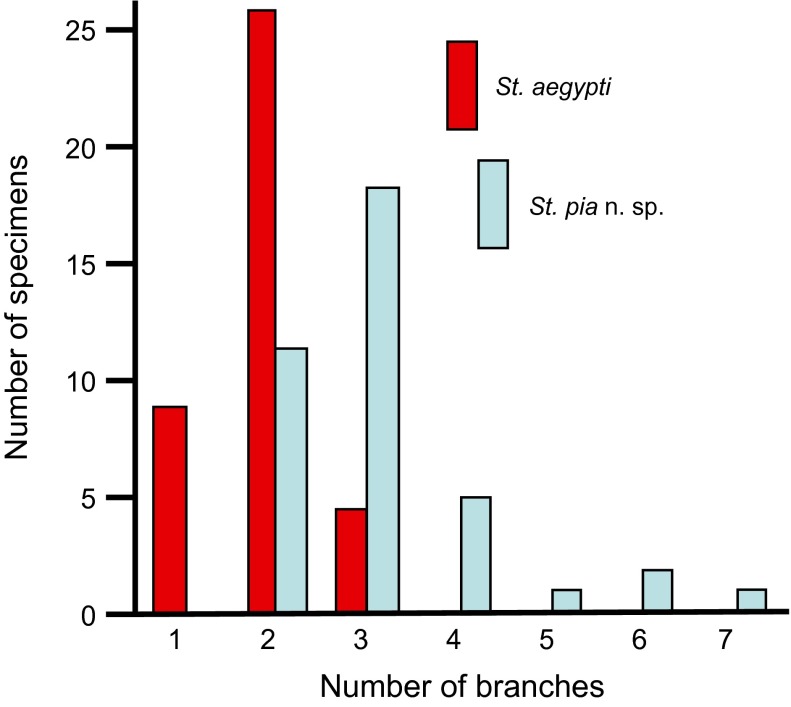
Distribution of the number of branches of seta 1-X of the fourth-instar larvae of *Stegomyia pia* n. sp. and *St*. *aegypti* (*n* = 39 and *n* = 38, respectively). When this number differed between the right and left sides of a single specimen, the highest number of branches was recorded.

**Figure 5.**
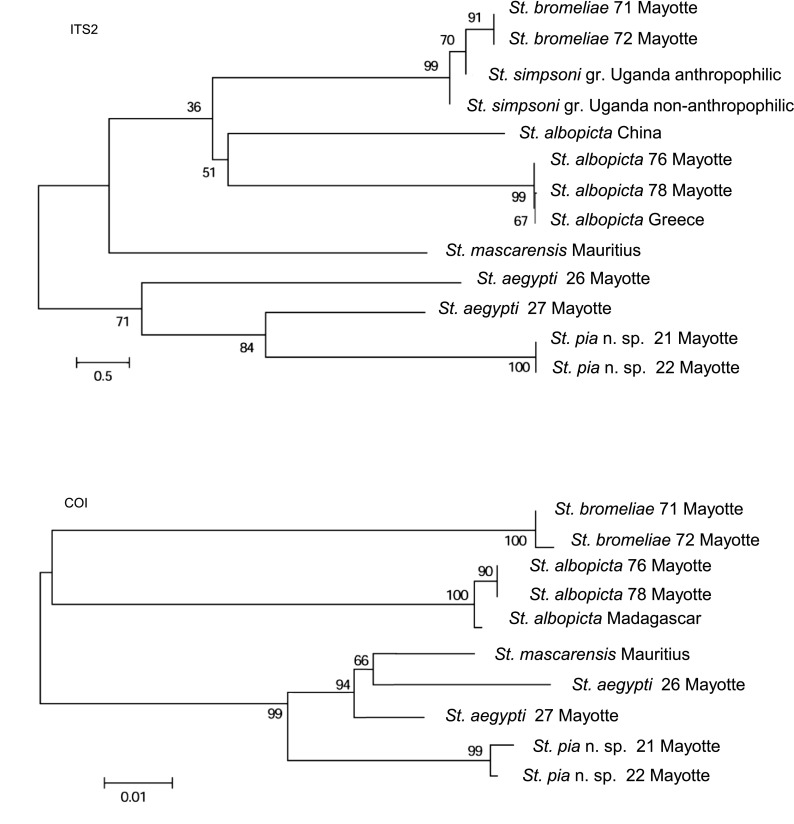
Neighbor-Joining trees based on *ITS2* and *COI* sequences. Bootstrap values were obtained after 1000 replications. See Table 3 for GenBank Accession numbers.

*Stegomyia bromeliae* is an important vector of yellow fever virus in East Africa [20].

**Figure 6.**
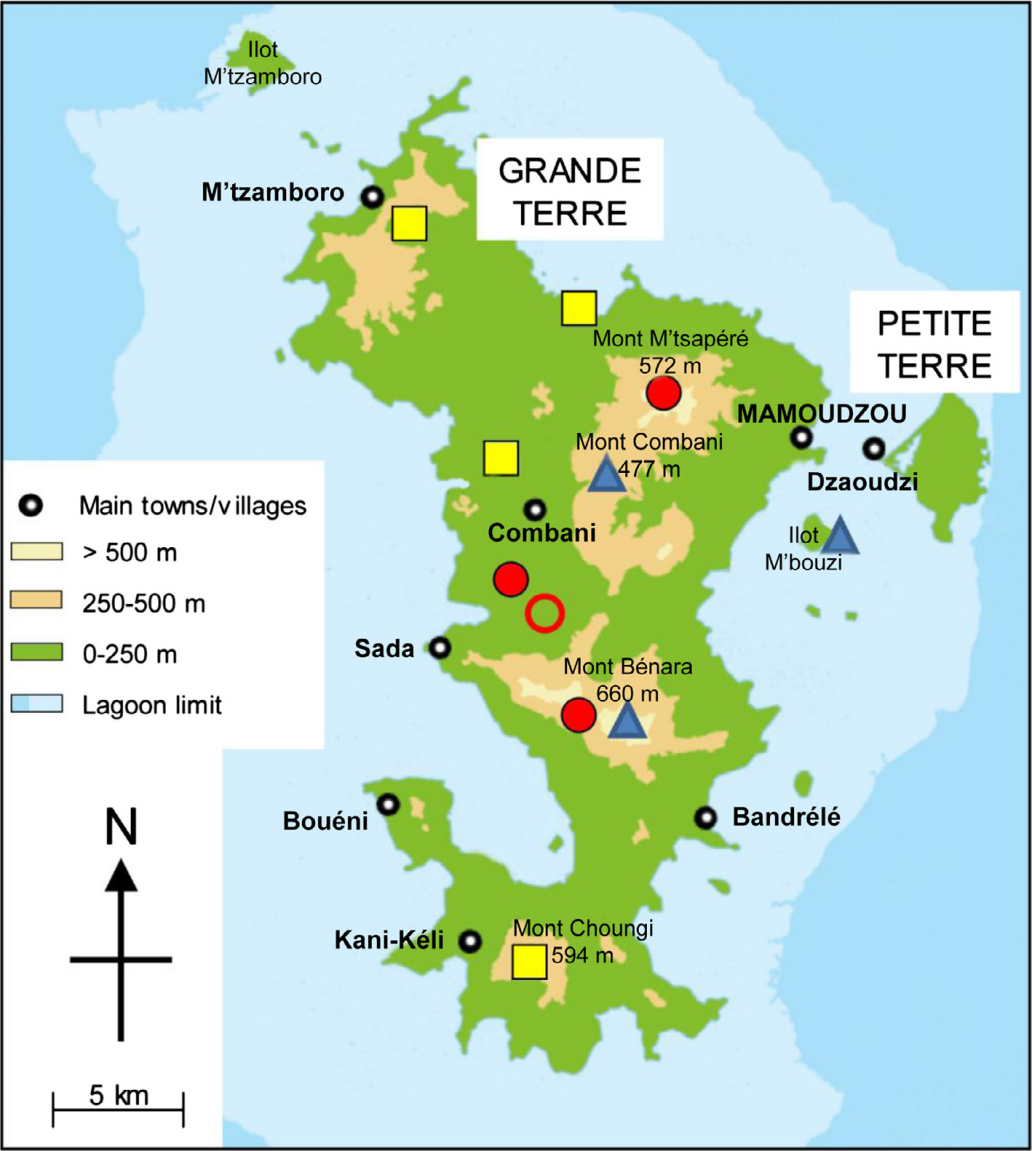
Schematic map of Mayotte with indication of the 11 sites where *Stegomyia pia* n. sp. has been recorded. Red disc, adults reared from pupae with confirmation by DNA sequencing; red circle, adults reared from pupae; yellow square, larvae with confirmation by DNA sequencing; blue triangle, larvae.

Despite the relative abundance of *St. pia* on Mayotte and its distinctive characters, this species has never been identified until now. This is puzzling and only tentative explanations can be proposed. First, the larval stages are morphologically similar to *St. aegypti*, which might explain some of the confusion. Second, we did not observe any males or females in our field collections. All adults were obtained in the insectary from larvae or more often from pupae collected in the field. When the first adult emerged, it was immediately clear that it belonged to a *Stegomyia* species that was hitherto unknown on Mayotte and the Comoros Archipelago. With these points in mind, we checked specimens in the Arim collection, obtained in the Comoros Archipelago and identified as *St. aegypti*. We found among about 30 *St. aegypti* one female of *St. pia* in a good state of preservation, collected by Dr. Alexis Grjebine on 19 February 1956 at larval stage in Coconi village on Mayotte. This is clear evidence that this species belongs to the Mayotte entomofauna but has been misidentified as *St. aegypti*. This location (12°50′024″S, 45°08′132″E) was added as an 11th site in the distribution map of the species (Figure 6).

**Figure 7.**
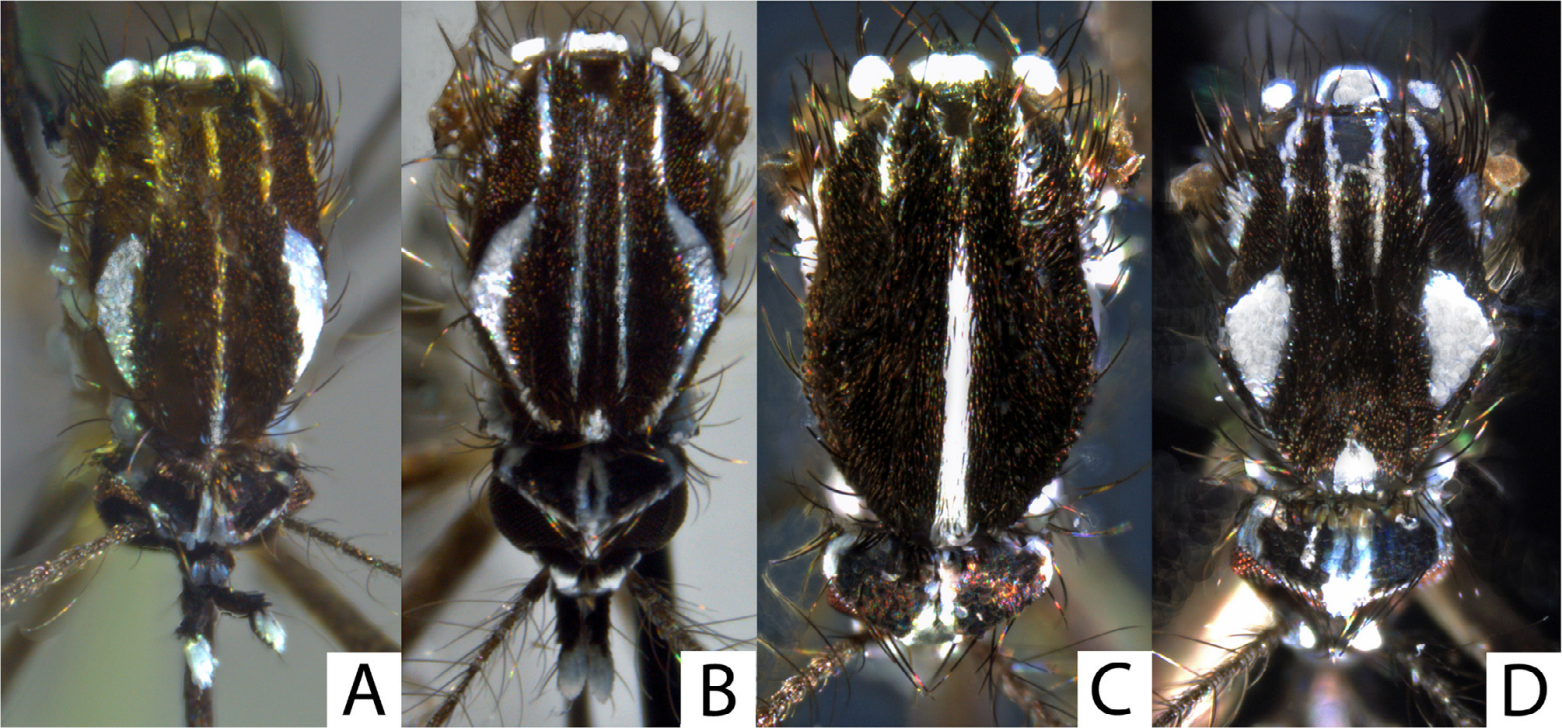
Microphotographs of the scutal ornamentation of the four species of *Stegomyia* present on Mayotte (dorsal views of females). A, *Stegomyia pia* n. sp. (specimen MY 628); B, *Stegomyia aegypti formosa* (specimen MY 007); C, *Stegomyia albopicta*; D, *Stegomyia bromeliae*.

The recognition of *St. pia* as a species new to science is clearly supported by a number of morphological characters in both males and females, and to a lesser extent in larvae. This recognition is reinforced by molecular data for all the gene sequences examined.

Due to the larval habitats utilised by *St. pia*, this species is of little consequence for the evaluation of stegomyian indices (aiming to estimate the density of potential vectors, an important criterion for risk estimation in public health) in anthropic and peri-domestic sites. However, immature stages of *St. pia* need to be taken into account on Mayotte when looking at relatively natural environments. Of interest is our observation of larval habitats in close proximity to villages in rural areas. The ability of this species to occupy woody zones in the periphery of towns needs further investigation.

*Stegomyia pia* is considered endemic on Mayotte. Further studies in the sister islands of The Union of the Comoros would be useful to determine if this species occurs elsewhere in this island group.

The bionomics of the adults are unknown, including the blood-feeding preference and longevity of females. However the potential vector role of *St. pia* may be hypothesised taking in mind the paramount importance of a number of *Stegomyia* species as vectors of many pathogens of medical and veterinary importance, including the West Nile, Yellow fever, St. Louis encephalitis, Dengue and Chikungunya viruses, as well as avian malaria such as *Plasmodium relictum* and several filarial nematodes such as *Dirofilaria immitis*.
